# Methanolic Extract from Aerial Parts of Artemisia Annua L. Induces Cytotoxicity and Enhances Vincristine-Induced Anticancer Effect in Pre-B Acute Lymphoblastic Leukemia Cells

**Published:** 2019-07-01

**Authors:** Pargol Mashati, Somayeh Esmaeili, Nasrin Dehghan-Nayeri, Davood Bashash, Mina Darvishi, Ahmad Gharehbaghian

**Affiliations:** 1 Department of Laboratory Hematology and Blood Bank, School of Allied Medical Sciences, Shahid Beheshti University of Medical Sciences, Tehran, Iran; 2 Tradintional Medicine and Materia Medica Research Center, School of Traditional Medicine, Shahid Beheshti University of Medical Sciences, Tehran, Iran; 3 Pediatric Congenital Hematologic Disorders Research Center, Shahid Beheshti University of Medical Sciences, Tehran, Iran

**Keywords:** Acute lymphoblastic leukemia, Vincristine, Extract, Apoptosis, Cytotoxicity

## Abstract

**Background:** Nowadays, remarkable attention has been drawn towards the effective therapeutic characteristic of natural products targeting cancerous cells. This study aimed to investigate the anti-cancer effect of *Artemisia annua *extract (AAE), a Chinese herbal medicine alone and in combination with a microtubule binding agent used in ALL treatment, vincristine (VCR), in B-Acute lymphoblastic leukemia (ALL) Nalm-6 and Reh cells.

**Materials and Methods:** Cytotoxic activity of AAE and VCR was determined using MTT assay in Nalm-6, and Reh cell lines and synergism was evaluated using the CompuSyn software. Caspase 3 activity and Annexin/PI staining were performed for apoptosis assessment. The expression level of apoptosis-related genes, caspase 3, Bax and Bcl-2 were determined using real time-PCR. One-way ANOVA and post hoc Tukey multiple comparisons were used for statistical analysis.

**Results:** Our findings revealed that a single administration of AAE exerted an anti-leukemic effect in both ALL-derived cells in a time- and dose-dependent manner. Interestingly, the growth inhibitory activity of the extract was more potentiated when combined with 0.1 and 1 nM VCR through caspase 3-dependent apoptosis. Moreover, real-time PCR analysis showed that VCR-induced cytotoxicity was augmented by AAE through alteration of Bax, and Bcl-2 mRNA expression.

**Conclusion:** Overall, owing to the nontoxic nature of AAE and its explicit role in enhancing VCR effectiveness, our study provided new insight into the development of a novel combinatorial approach in ALL using natural herbs. The practical implication of the research requires further investigation through clinical trials, opening avenues for forthcoming treatment improvements.

## Introduction

 Acute lymphoblastic leukemia (ALL) affects both children and adults with a peak occurrence at 2 to 5 years of age^1^^,^^2^^,^^3^. Despite substantial improvements in treatment of ALL patients, the relapse rate is still considered as a major challenge in these people^4^^,^^5^. VCR, a microtubule binding agent which arrests dividing cells in metaphase, is a common chemotherapeutic drug used for ALL treatment. Noteworthy, compelling body of evidence has indicated, thus far, a variety of adverse effects for this drug such as neuropathy, drug resistance and immune system suppression ^6^. It is not surprising that increasing demands for new strategies to overcome these adverse effects, such as using multicomponent therapy or combination therapy, have attracted tremendous attentions. The herb extracts, served as potential synergistic agents due to their complex mixture of phytochemicals, are applied as reliable candidates for therapeutic interventions^7^^,^^8^. *Artemisia*, which belongs to Asteraceae (Compositae) family^9^, is rich in main chemical components, including coumarins, flavonoids, sterols, monoterpenes, polyacetylenes and sesquiterpene lactones^10^^, ^^11^. Growth inhibitory effects of various species of the genus *Artemisia* on human cell lines have been reported in several recent studies. Tayarani-Najaran Z et al. indicated that dichloromethane (CH_2_Cl_2_) and petroleum ether (PE) extracts of *Artemisia ciniformis* exerted cytotoxic effects on leukemic cell lines ^12^. Moreover, CH_2_Cl_2 _extracts of *Artemisia diffusa*, *Artemisia ciniformis *and* Artemisia santolina* have been demonstrated to induce growth inhibitory effect on adenocarcinoma cells ^13^. Furthermore, considerable anti-cancer effects of artemisinin, an isolated compound from AAE as well as its derivatives, have been studied in leukemic cell lines ^14^. In the light of these findings, we aimed to investigate the cytotoxicity of total methanolic extract of* Artemisia annua*. In this study, for the first time, we evaluated the inhibitory effect of AAE on the cell survival rate in two distinct acute lymphoblastic leukemia cell lines, Nalm-6 and Reh cells. In addition, we evaluated whether treated leukemic cells with this extract could enhance the sensitivity of cells to the cytotoxic effect of VCR.

## MATERIALS AND METHODS

**Chemicals**

VCR was purchased from Sigma-Aldrich Co. LLC. RPMI 1640 was purchased from Gibco (Invitrogen, Gibco, USA). Fetal bovine serum (FBS) was purchased from PAN-Biotech GmbH. Dimethyl sulphoxide (DMSO) and 3-[4,5-dimethylthiazol-2-yl]5554 (14)-2,5-diphenyltetrazolium bromide were purchased from Roth (Germany). 

**Plant extract**

AAE was collected from Golestan, Kalale province, located in the north of Iran. A voucher specimen was deposited at the Herbarium of Traditional Medicine and Materia Medica Research Center, Shahid Beheshti University of Medical Sciences, Tehran, Iran (No. 5421). The powder of aerial parts of AAE was macerated in methanol, and left on a shaker for a day at room temperature. The filtrate was evaporated to dryness and used for evaluation. 100 mg of the extract was dissolved in 1 ml DMSO, and then desired concentrations of the extract were prepared by dissolving in DMSO, reducing the final concentration of DMSO to 0.01%.

**Cell lines and culture conditions**

ALL cell lines Nalm-6 and Reh were obtained from Pasteur Institute of Iran and peripheral blood mononuclear cells (PBMCs) were prepared by Ficoll–Hypaque gradient centrifugation stimulated by phytohemagglutinin (PHA). Cells were cultured in RPMI 1640 medium supplemented with 10% FBS at 37 ^֯^C in a humidified incubator with 95% air and 5% CO_2_. 

**Cell viability assay**

Nalm-6 and Reh cell lines at the density of 3.0 × 10^4 ^cells/well and normal lymphocytes at the density of 7.0 × 10^4^ cells/well were seeded in 96-well plates. The cells were treated with AAE, VCR and the combination of two drugs for 48 and 72 h. Cells were grown in 100 µl RPMI 1640 medium supplemented with 10% FBS. After the incubation period, 20 µl of MTT (0.5mg/ml) was added and the plates were allowed to incubate further for 4h at 37 ^֯^C. The cell culture supernatant was removed, 100 µl of DMSO was added to each well and the plate was shaken for 10 min. The absorbance of each well was detected at 570 nm on an ELISA plate reader. IC_50_ was defined as the concentration of drug that inhibited cell growth by 50% compared to untreated controls. Each experiment was carried out in triplicate. To measure the extent of interaction between AAE and VCR, these data were analyzed by CompuSyn software 1.0 (ComboSyn Incorporated.) to study the synergism/antagonism effect and also to evaluate the benefits of combined treatment compared to individual treatment. 

**Flow cytometry analysis**

Nalm-6 and Reh cells (0.6 × 10^6^) were exposed to various concentrations of AAE and the combination of the extract and VCR for 48 h. Non-treated cells at 0 h were used as the control group. The plates were incubated for 48h. Untreated cells were used as the control group. After 48 h incubation, cells were washed in PBS for once, then once in 1x Binding Buffer. The cells resuspended in 1x Binding Buffer. Then 5 mL of fluorochrome-conjugated Annexin V (Annexin V/FITC kit, eBioscience, USA) were added to 100 mL of the cell suspension and after 15 minutes’ incubation at room temperature, 16 mL of Propidium Iodide (PI) staining solution was added to each microtube. The tubes were vortexed and incubated for 15 minutes at room temperature in the dark and then were analyzed by flow cytometry (Partec PAS III, Germany) and illustrated using FloMax software. All experiments in this assay were accomplished in triplicate.

**RNA isolation and quantitative real-time PCR**

To carry out qRT-PCR, the cells were treated with AAE and VCR and then incubated for 48 h. Total RNA was extracted from each well using the Hybrid-R RNA purification kit (GeneAll, Korea). The quantity of RNA samples was determined by nanodrop instrument (Nanodrop TM 2000 Spectrophotometer, Thermo Scientific, USA). RNA from each sample went through reverse transcription, and first-strand cDNA was synthesized (Thermo Scientific, USA). The prepared cDNA, used as a template for polymerase chain reaction (PCR) amplification, was subjected to Real-time PCR analysis, applying SYBER Premix Ex Taq (Tli RNase H plus) kit (Takara Biomedical Technology, Japan) in Rotor-Gene Q Real-Time PCR System (Qiagen, Valencia, CA), with the Rotor-Gene Q Series Software. To normalize the expression of the target genes, the expression of the ABL gene was used as an endogenous control. The mean Ct of caspase 3, Bax and Bcl-2 genes were calculated from triplicate measurements and normalized with the mean Ct of the ABL gene. Melting curves were evaluated to validate caspase 3, Bax and Bcl-2 and ABL single PCR product. The relative quantification of target genes was determined using the Pfaffl method^15^. The sequences of the primers are presented in Table 1.

**Caspase 3 activity assay**

The activity of caspase 3 was determined by caspase colorimetric assay kit according to the manufacturer’s protocol (Abcam, MA, USA) (ab39401). Briefly, 1 × 10^6^ cells were treated with different concentrations and different combinations of drugs for 48 h, and they were collected, washed with ice-cold PBS and lysed in a lysis buffer. Each cell lysate was centrifuged at 10000 g for 1 min, and the supernatant was collected. After protein quantification using Bicinchoninic acid assay (BCA) (Sigma-Aldrich, St Louis, MO, USA), 50 μl of each sample were added to 96 well plate and 50 μl of 2× reaction buffer containing 10 mM DTT and 4 mM caspase 3 substrate (DEVD-p-NA) were added to 200 μg protein from each sample and then incubated at 37 °C for 4, 12 and 24 h. 50 μl of reaction buffer was used in background wells. The absorbance of the final reaction mixture was measured at the 405 nm wavelength. The amounts of caspase enzymatic activities in cell lysates were straightly relative to the color reaction.

**Statistical analysis**

The SPSS 23 software was used for data analysis. One-way ANOVA and post hoc Tukey multiple comparisons were used for statistical analysis. Results were presented as mean ± SE (SEM). A value of P < 0.05 was considered significant.

**Table1:** Genes and oligonucleotide primers for quantitative real-time RT-PCR

Gene	Accession number	Forward primer (5′−3′)	Reverse primer (5′−3′)
**ABL**	NM_080104	CTTCTTGGTGCGTGAGAGTGAG	GACGTAGAGCTTGCCATCAGAAG
**CASP-3**	NM_032991	AAATACCAGTGGAGGCCGACT	TCAGCATGGCACAAAGCGAC
**BAX**	NM_138761	CATGGAGCTGCAGAGGATGATTG	CCAGTTGAAGTTGCCGTCAGAA
**BCL-2**	NM_000633	TGATGGGATCGTTGCCTTATGC	TCAGTCTACTTCCTCTGTGATGTTGTA

ABL (Abelson murine leukemia viral oncogene homolog), CASP-3 (cysteine-aspartic proteases 3), BCL-2 (B-cell lymphoma 2), BAX (BCL-2 associated X protein)

## Results

**Cytotoxic effects of AAE and VCR as single agents and in combination on Nalm-6 and Reh cells**

**Figure 1:**
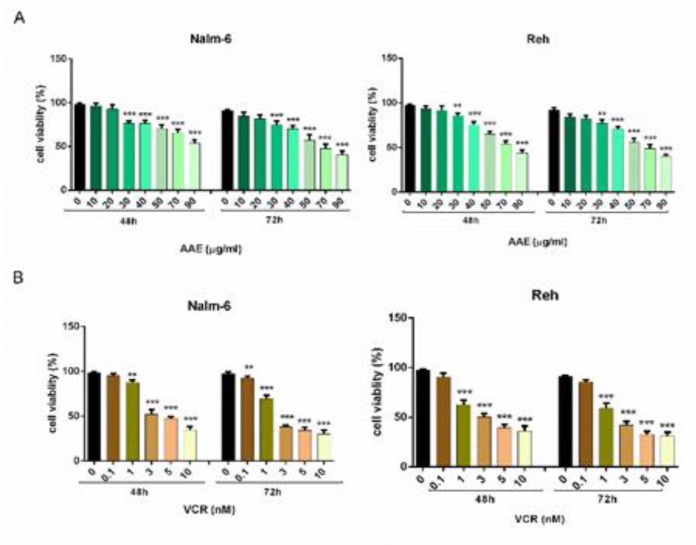
(A) Effect of individual treatment of AAE (10 to 90 μg/ml) on Nalm-6 and Reh cells after 48 h and 72 h (B) Effect of individual treatment of VCR (0.1 to 10 nM) on Nalm-6 and Reh cells after 48 h and 72 h. The cell viability was determined by MTT assay. Each bar represents the mean ± SD (n=3). *P< 0.05, **P< 0.01, ***P< 0.001 vs control; ^a ^P< 0.05, ^c ^P<0.001 vs VCR (one-way ANOVA and post hoc Tukey multiple comparison test).

**Figure 2:**
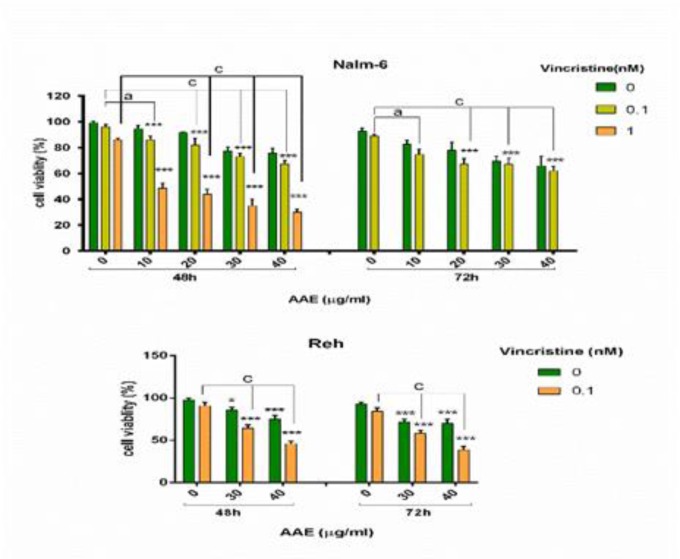
Effect of co-treatment of various concentrations of AAE (10, 20, 30, 40 μg/ml) and VCR (0.1 and 1 nM) on Nalm-6 (A) and Reh (B) cells after 48 h and 72 h. The cell viability was determined by MTT assay. Each bar represents the mean ± SD (n=3). *P< 0.05, **P< 0.01, ***P< 0.001 vs control; ^a ^P< 0.05, ^c ^P<0.001 vs VCR (one-way ANOVA and post hoc Tukey multiple comparison test).

To determine the cytotoxic effect of AAE, Nalm-6 and Reh cell lines were treated with various concentrations of AAE (10, 20, 30, 40, 50, 70, 90 µg/ml) and VCR (0.1, 1, 3, 5, 10 nM) for 48 and 72 h. As shown in Fig. 1, treatment of the cells with the extract exhibited time- and dose-dependent effect on both cell lines. In Reh cells, 70 µg/ml of AAE caused cell viability of 52 % after 48 h and 47 % following 72 h treatment (Figure 1A). Interestingly, AAE induced limited anti-tumor activity in PBMCs (IC_50_ ˃ 100 µg/mL) compared to leukemic cells (Data not shown). Moreover, to investigate the effect of the extract on increasing sensitivity of leukemic cells to VCR, we co-treated both cell lines with AAE and VCR (Table 2) (Figure 2). We observed a marked decrease in viability of Reh cells when the cells were treated with 0.1 nM VCR plus 40 µg/ml AAE as displayed in Figure 2B. Also, in Nalm-6 cells, the combination of 1 nM VCR and 20 µg/ml of the extract resulted in a noticeable reduction in cell viability (Figure 2A). These combinatorial doses were selected for further experiments.

**Table 2:** The combination indices (CI) for the growth inhibitory effects of combined treatments

Doses	0.1VCR+30AAE	0.1VCR+40AAE	1VCR+10AAE	1VCR+20AAE
**Nalm-6**	0.74 ± 0.07	0.82 ± 0.04	0.13 ± 0.05	0.23 ± 0.05
**Reh**	0.61 ± 0.01	0.57 ± 0.007	ND	ND

Data are expressed as mean ± SD. CI less than 0.9 indicates synergism.

ND: not determined

**Induction of apoptosis in Nalm-6 and Reh cells treated with AAE and VCR**

**Figure 3:**
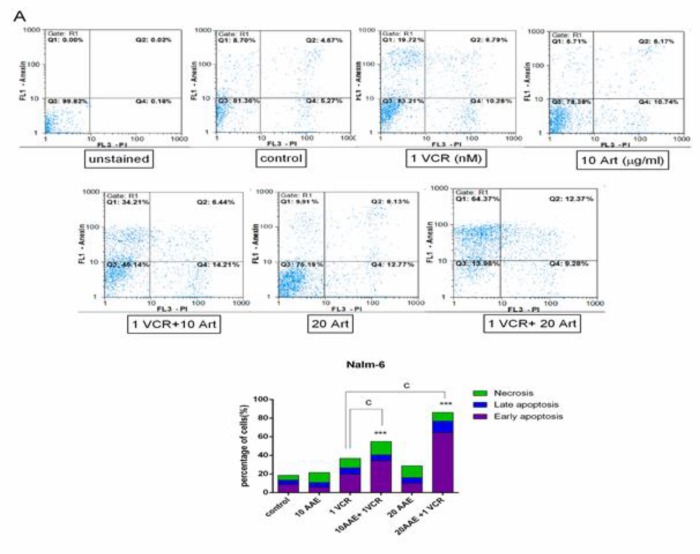
Nalm-6 Cells were treated with 10 and 20 µg/ml AAE alone and in combination with 1 nM VCR. Annexin V-FITC/PI double staining discriminates the live cells (Annexin V−/PI−; bottom left quadrant), early apoptotic cells (Annexin V+/PI−; upper right quadrant), late apoptotic or necrotic cells (Annexin V+/PI+; upper right quadrant), and dead cells (Annexin V−/PI+; bottom left quadrant). **P< 0.01, ***P< 0.001 vs control; ^c^P< 0.001 vs VCR (one-way ANOVA and post hoc Tukey multiple comparison test).

**Figure 4:**
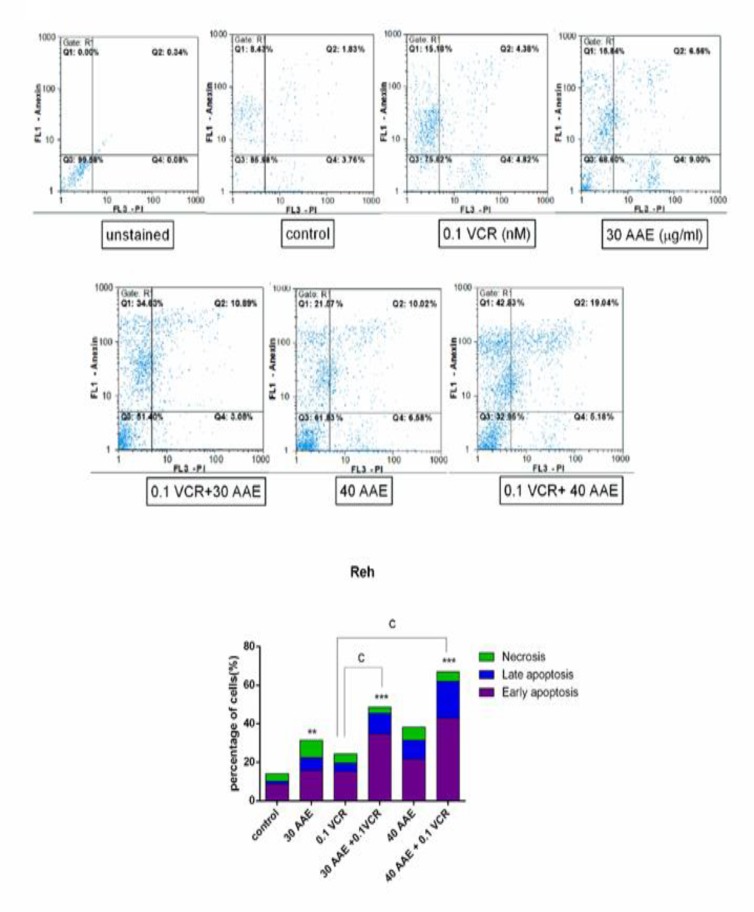
Reh cells were treated with 30 and 40 µg/ml AAE alone and in combination with 0.1 nM VCR. Annexin V-FITC/PI double staining discriminates the live cells (Annexin V−/PI−; bottom left quadrant), early apoptotic cells (Annexin V+/PI−; upper right quadrant), late apoptotic or necrotic cells (Annexin V+/PI+; upper right quadrant), and dead cells (Annexin V−/PI+; bottom left quadrant). **P< 0.01, ***P< 0.001 vs control; ^c^P< 0.001 vs VCR (one-way ANOVA and post hoc Tukey multiple comparison test).

To determine whether the inhibitory effects of AAE as a single agent and in combination with VCR could be attributed to the induction of apoptosis, we decided to evaluate the effectiveness of the treatment on the modulation of phosphatidylserine externalization using Annexin V/PI staining. As is evident in Figure 4, 40 µg/ml of the extract induced early-apoptosis in Reh cells, which was more noticeable than a single treatment of AAE in Nalm-6. In agreement with MTT results, simultaneous treatment of AAE and VCR resulted in the remarkable promotion of both Annexin V and Annexin V/PI double positive cells in Reh cells (P< 0.001; Figures 3 and 4). As presented in Figure 4, the combination of 0.1 nM VCR and 40 µg/ml AAE resulted in 42.83% early apoptosis and 19.04% late apoptosis in Reh cells following 48 h treatment. It is noteworthy that only 19.72% and 15.18 % early apoptosis were observed in Nalm-6 and Reh cells, respectively, following treatment with VCR alone.

**mRNA expression levels of caspase 3, Bax and Bcl-2 genes** **in Nalm-6 and Reh cells treated with AAE and VCR **

**Figure 5:**
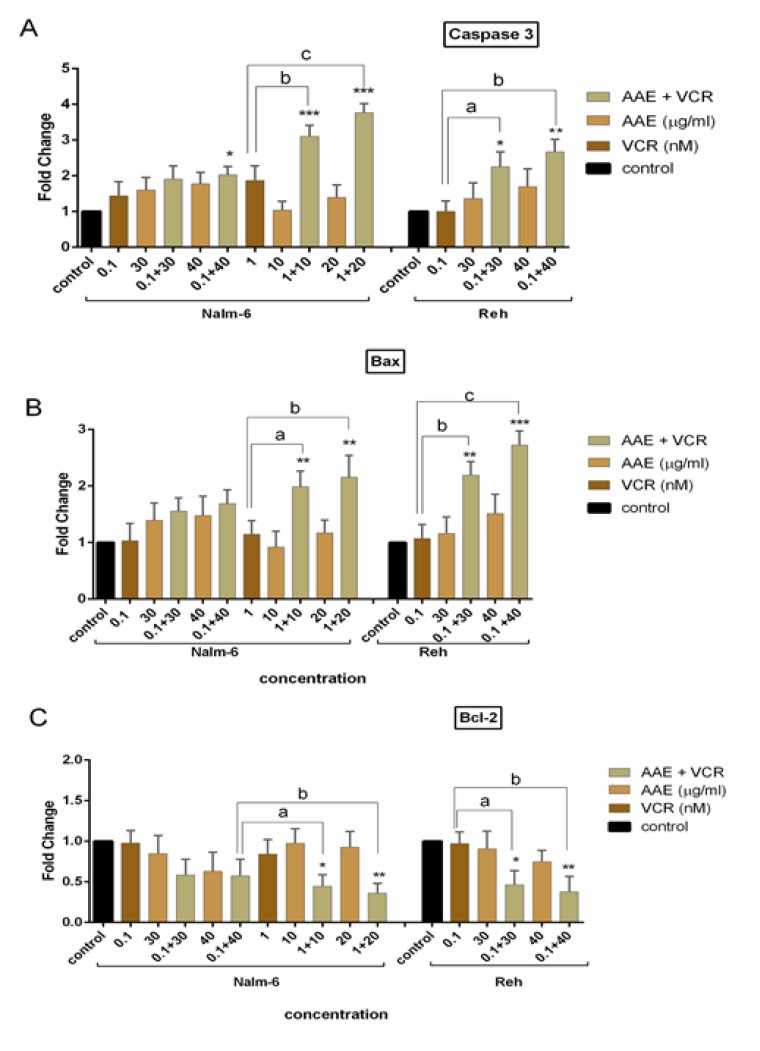
Quantitative real time-PCR analysis of (A) caspase 3, (B) Bax, (C) Bcl-2 mRNA expression levels in Nalm-6 and Reh cells. Total mRNA was detected by Real-time Quantitative PCR analysis. ABL served as an internal control. Each bar represents the mean ± SD (n=3). *P< 0.05, **P< 0.01, ***P< 0.001 vs control; ^A^P <0.05, ^b^P< 0.01, ^c^P< 0.001 vs VCR (one-way ANOVA and post hoc Tukey multiple comparison test).

Having established the apoptotic effects of the extract in combination with VCR, we carried out a real time-PCR analysis to investigate the role of mRNA expression of apoptotic genes in both cell lines. We found that 40 µg/ml of the extract increased the mRNA expression level of caspase 3 and Bax, as the most important genes involved in apoptotic pathway in both cell lines, which were coupled with explicit suppression effect on the transcriptional activity of the anti-apoptotic-related gene, Bcl-2, in both Nalm-6 and Reh cells (Figure 5). Moreover, the synergistic treatment of the cells resulted in more substantial modification in the expression of apoptotic genes with similar results in both cell lines.

As depicted in Figures 5A and B, caspase 3 and Bax were increased by nearly 2.6- and 2.72-fold when Reh cells were co-treated with AAE and VCR, respectively.

**Effects of AAE and VCR on activation of caspase-3 in Nalm-6 and Reh cells**

**Figure 6:**
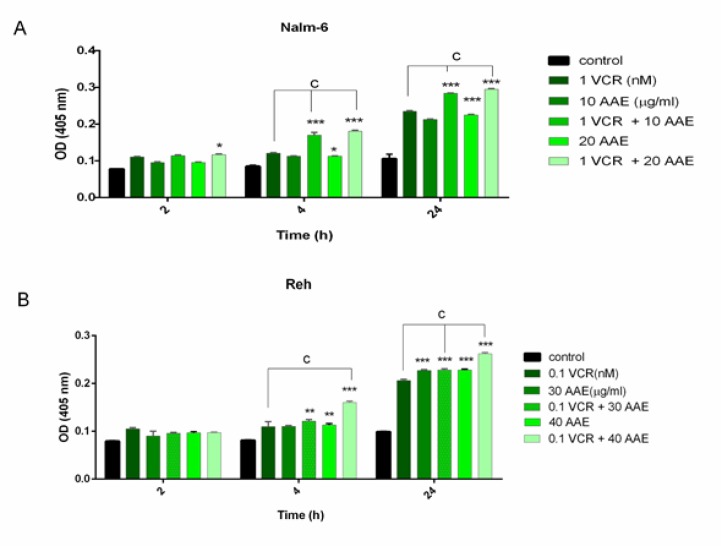
Quantification of caspase 3 activity in Nalm-6 and Reh cells. (A) Treatment of Nalm-6 cells with 10 and 20 μg/ml AAE alone and in combination with 1 nM VCR. (B) Treatment of Reh cells with 30 and 40 μg/ml AAE alone and in combination with 0.1 nM VCR. Each bar represents the mean ± SD (n=3). *P< 0.05, **P< 0.01, ***P< 0.001 vs control; ^a ^P <0.05, ^b ^P< 0.01, ^c ^P< 0.001 vs VCR (one-way ANOVA and post hoc Tukey multiple comparison test).

To ascertain whether the induction of apoptosis is mediated through the caspase-dependent cascade, the enzymatic activation of caspase 3 was investigated as an executioner enzyme of apoptosis. The resulting data revealed that the activity of caspase 3 was increased more notably in synergistic experiments compared to either treatment alone. As presented in Figure 6, 0.1 and 1 nM VCR induced a modest increase in activation of caspase 3, whereas, in combination with AAE, it was elevated more significantly in Nalm-6 and Reh cells.

## Discussion

 Since time immemorial, medicinal herbs have been broadly used as a primary source for the treatment of cancer, either applied individually or as adjuvant with conventional treatments^16^. Unlike chemotherapy drugs, plant extracts, owing to their complex multi-component nature, have achieved prominent therapeutic efficacy targeting molecules in two or more biological pathways responsible for cancer progression^17^^,^^18^. Naturally occurring AAE, a prominent Chinese therapeutic plant mostly distributed in Asia and some parts of Africa, has been shown to possess growth inhibitory properties in various cell lines. A recent report revealed that AAE reduced the survival rate of T-cell leukemic cells in a dose- and time-dependent manner^19^. Another study conducted by Worku et al. reported the cytotoxic effect of this extract on solid tumor cell lines such as prostate cancer cells^20^. Moreover, Eun Ji Kim indicated that AAE could exert an apoptotic effect on colon cancer cells^21^. In an effort to investigate the potential therapeutic value of AAE, for the first time, we aimed to evaluate the restrictive effect of methanolic extract from aerial parts of *Artemisia annua* on both Nalm-6 and Reh cell lines alone and in combination with VCR.

The results obtained in our study demonstrated that a single administration of AAE reduced the viability of B-ALL-derived cells in a time- and dose-dependent manner. We evaluated the IC_50 _of the extract as 70 and 90 µg/ml in Reh and Nalm-6 cell lines, respectively; however, the significant finding of our study was that AAE synergistically potentiated VCR-induced cytotoxicity in ALL-derived cells. 

Our time- and concentration-dependent synergistic experiments revealed that metabolic activity was considerably hindered upon exposure of Reh cells to 0.1 nM VCR AAE in combination with 40 µg/ml AAE. We noticed a similar inhibitory effect on Nalm-6 cells after co-treatment of 1 nM VCR and 20 µg/ml AAE followed by 48 h incubation. Our findings also delineated that AAE augments VCR-induced apoptosis as evidenced by increased externalization of phosphatidylserine compared to the single effect of VCR. The finding was further strengthened by meaningful alteration in the transcriptional level of apoptotic genes when leukemic cells were co-treated with AAE and VCR as compared with either agent alone. Moreover, the activation of caspase 3 was more evident in synergistic treatment in both cell lines. In a similar study, Darvishi et al. showed that combinational treatment of VCR and *Juniperus excelsa* led to increased Bax/Bcl2 ratio and activation of caspase 3^22^. Overall, our findings support the idea that AAE in combination with VCR might be a useful strategy in the development of novel combination therapy in ALL. 

## CONCLUSION

 Taken together, our study demonstrated that AAE either as a single agent or in combination with VCR could display significant cytotoxicity against ALL cell lines, Nalm-6, and Reh cells. Our study suggests that the administration of this compound in combination with VCR allow lower concentrations of the chemotherapeutic drug to be used, resulting in less nonspecific toxicity in ALL treatment. However, the investigation through the clinical setting could further validate our study results and determine the efficacy of this approach.

## References

[1] Inaba H, Greaves M, Mullighan CG (2013). Acute lymphoblastic leukaemia. Lancet.

[2] Dehghan-Nayeri N, Eshghi P, Pour KG (2017). Differential expression pattern of protein markers for predicting chemosensitivity of dexamethasone-based chemotherapy of B cell acute lymphoblastic leukemia. Cancer Chemother. Cancer Chemother Pharmacol.

[3] Nikbakht M, Jha AK, Malekzadeh K (2017). Aberrant promoter hypermethylation of selected apoptotic genes in childhood acute lymphoblastic leukemia among North Indian population. Exp Oncol.

[4] Arpe ML, Rørvig S, Kok K (2015). The association between glucocorticoid therapy and BMI z-score changes in children with acute lymphoblastic leukemia. Support Care Cancer.

[5] Bahmani F, Esmaeili S, Bashash D (2018). Centaurea albonitens extract enhances the therapeutic effects of Vincristine in leukemic cells by inducing apoptosis. Biomed Pharmacother..

[6] Chao MW, Lai MJ, Liou JP (2015). The synergic effect of vincristine and vorinostat in leukemia in vitro and in vivo. J Hematol Oncol..

[7] Lahlou M (2013). The success of natural products in drug discovery. Pharmacol Pharm.

[8] Yang Y, Zhang Z, Li S (2014). Synergy effects of herb extracts: pharmacokinetics and pharmacodynamic basis. Fitoterapia..

[9] Mucciarelli M, M Maffei, Wright CW (2002). Artemisia. Medicinal and aromatic plants-industrial profiles. Artemisia.

[10] Tan RX, Tang HQ, Hu J (1998). Lignans and sesquiterpene lactones from Artemisia sieversiana and Inula racemosa. Phytochemistry.

[11] Bora KS, Sharma A (2011). The genus Artemisia: a comprehensive review. Pharm Biol.

[12] Tayarani-Najaran Z, Hajian Z, Mojarrab M (2014). Cytotoxic and apoptotic effects of extracts of Artemisia ciniformis Krasch and Popov ex Poljakov on K562 and HL-60 cell lines. Asian Pac J Cancer Prev.

[13] Taghizadeh Rabe SZ, Mahmoudi M, Ahi A (2011). Antiproliferative effects of extracts from Iranian Artemisia species on cancer cell lines. Pharm Biol.

[14] Fox JM, Moynihan JR, Mott BT (2016). Artemisinin-derived dimer ART-838 potently inhibited human acute leukemias, persisted in vivo, and synergized with antileukemic drugs. Oncotarget.

[15] Pfaffl MW (2001). A new mathematical model for relative quantification in real-time RT–PCR. Nucleic Acids Res.

[16] Li, F-S, J.-K. Weng (2017). Demystifying traditional herbal medicine with modern approach. Nature plants.

[17] Leonti M, R Verpoorte (2017). Traditional Mediterranean and European herbal medicines. Journal of ethnopharmacology..

[18] Foo JB, Saiful Yazan L, Tor YS (2015). Induction of cell cycle arrest and apoptosis by betulinic acid-rich fraction from Dillenia suffruticosa root in MCF-7 cells involved p53/p21 and mitochondrial signalling pathway. J Ethnopharmacol..

[19] Singh NP, Ferreira JF, Park JS (2011). Cytotoxicity of ethanolic extracts of Artemisia annua to Molt-4 human leukemia cells. Planta Med.

[20] Worku N, Mossie A, Stich A (2013). Evaluation of the in vitro efficacy of Artemisia annua, Rumex abyssinicus, and Catha edulis Forsk extracts in cancer and Trypanosoma brucei cells. ISRN Biochem.

[21] Kim EJ, Kim GT, Kim BM (2017). Apoptosis-induced effects of extract from Artemisia annua Linné by modulating PTEN/p53/PDK1/Akt/signal pathways through PTEN/p53-independent manner in HCT116 colon cancer cells. BMC Complement Altern Med.

[22] Darvishi M, Esmaeili S, Dehghan-Nayeri N (2015). Anticancer effect and enhancement of therapeutic potential of Vincristine by extract from aerial parts of Juniperus excelsa on pre-B acute lymphoblastic leukemia cell lines. J Appl Biomed.

